# Predatory Bacteria in the Treatment of Infectious Diseases and Beyond

**DOI:** 10.3390/idr16040052

**Published:** 2024-07-25

**Authors:** Konstantinos Alexakis, Stella Baliou, Petros Ioannou

**Affiliations:** 1 German Medical Institute, Limassol 4102, Cyprus; a.konstantin91@gmail.com; 2 School of Medicine, University of Crete, 71003 Heraklion, Greece

**Keywords:** predatory bacteria, *Bdellovibrio bacteriovorus*, antimicrobial resistance, *Micavibrio aeruginosavorus*

## Abstract

Antimicrobial resistance (AMR) is an increasing problem worldwide, with significant associated morbidity and mortality. Given the slow production of new antimicrobials, non-antimicrobial methods for treating infections with significant AMR are required. This review examines the potential of predatory bacteria to combat infectious diseases, particularly those caused by pathogens with AMR. Predatory bacteria already have well-known applications beyond medicine, such as in the food industry, biocontrol, and wastewater treatment. Regarding their potential for use in treating infections, several in vitro studies have shown their potential in eliminating various pathogens, including those resistant to multiple antibiotics, and they also suggest minimal immune stimulation and cytotoxicity by predatory bacteria. In vivo animal studies have demonstrated safety and efficacy in reducing bacterial burden in various infection models. However, results can be inconsistent, suggesting dependence on factors like the animal model and the infecting bacteria. Until now, no clinical study in humans exists, but as experience with predatory bacteria grows, future studies including clinical studies in humans could be designed to evaluate their efficacy and safety in humans, thus leading to the potential for approval of a novel method for treating infectious diseases by bacteria.

## 1. Introduction

The application of hand hygiene and the discovery of antibiotics have revolutionized medicine by reducing the likelihood, morbidity, and mortality of infectious diseases. However, soon after the introduction of antimicrobial treatment in infectious diseases, it became evident that antimicrobial resistance (AMR) would quickly arise [1,2]. The antimicrobial pipeline provided an adequate number of antimicrobials for many decades. Still, in the last decades, AMR has evolved as a significant problem of public health importance. Meanwhile, at the same time, the production of novel antimicrobials has been stalled [3]. More specifically, there has been a significant increase in infections by multi-drug-resistant (MDR), extensively drug-resistant (XDR), and pan-drug-resistant (PDR) pathogens that often have very limited options for antimicrobial treatment [4,5,6].

Infections by pathogens with AMR often have a worse prognosis and require prolonged intravenous antimicrobial treatment and longer hospital stays [6,7]. Few new antimicrobials have been introduced in clinical practice in recent years, and most commonly, they belong to previously known antimicrobial classes [8,9,10,11]. Thus, introducing novel methods for treating pathogens, especially those with significant AMR, may be valuable for reducing morbidity and mortality from infections caused by such microorganisms. Such examples may include bacteriophages, antimicrobial peptides, and applications of nanotechnology [12,13,14].

Using living predatory bacteria that cannot harm humans but can fight pathogens could also be an alternative for treating infectious diseases in humans. The present study aimed to critically review the evidence regarding the use of predatory bacteria in the fight against infectious diseases and beyond.

## 2. Biology of Predatory Bacteria

Predatory bacteria are a unique group of bacteria that possess the ability to invade and consume other bacterial species. They utilize a range of strategies, such as secretion of lytic enzymes and direct contact feeding, to prey on neighboring bacteria. For example, members of the *Bdellovibrionales* can attach to other bacteria, penetrate their membrane, and reside in their cytoplasm, allowing them to consume cellular parts of their targets, leading to the production of daughter cells, lysis, and cell death of their bacterial target [15,16]. These microorganisms are abundant in the environment and can be found naturally in many different settings, such as in soil, rivers, open sea, wastewater treatment plants, and even in animals [17,18,19,20,21,22,23,24,25,26].

Predatory bacteria have different mechanisms of predation and are often categorized based on their lifestyle [20]. For example, wolf-pack predators are opportunistic predators capable of axenic growth but can predate on other bacteria in some circumstances. Some examples include *Lysobacter* spp. and Myxobacteria, such as *Corallococcus* spp., *Pyxidicoccus* spp., and *Myxococcus* spp. [27,28]. These bacteria are considered to require the presence of many predators to allow adequate lysis of their targets, with the lysis being considered to occur through membrane vesicle secretion, allowing the release of enzymes and metabolites that act on their bacterial targets [29,30,31]. However, evidence exists that at least some of these types of predatory bacteria can cause bacterial target lysis on an individual basis [32]. These predatory bacteria can prey on a wide range of bacterial species given their ability to cause lysis of their target in a non-specific way through secretion of antibacterial substances and can also survive in the absence of bacterial targets and form biofilms [28,33,34].

Cytoplasmic predatory bacteria include only *Daptobacter*. This bacterium is a facultative predator, can grow in an axenic manner in the absence of prey, and divides by binary fission [35]. When acting as a predator, it penetrates the bacterial membranes and enters the cytoplasm of its prey, members of *Chromatiaceae* [35].

Epibiotic predators include bacteria such as *Pseudobdellovibrio exovorus*, *Vampirovibrio chlorellavorus*, *Vampirococcus* (Candidate Phyla Radiation), and *Micavibrio aeruginosavorus* [20,36,37,38,39]. These are obligate predators, meaning that their survival and growth depend on the presence of prey bacteria [20]. These predatory bacteria can consume other bacteria by attaching to their surface, forming pores after degrading their membrane at the point of attachment, degrading and consuming their macromolecules, and septation and binary fission of the predatory bacteria [36].

**Figure 1:**
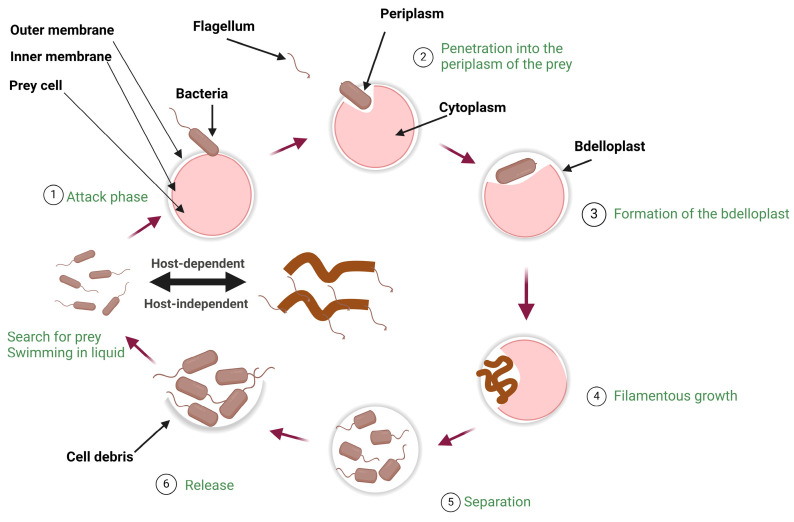
Shown is the life cycle of intraperiplasmic predatory bacteria. Predatory bacteria attack their prey, penetrate the periplasm, form the bdelloplast, resemble filaments, and grow intracellularly and are eventually released by killing their bacterial prey and releasing their progeny in search of another target bacterial cell. The image was created with Biorender.

Finally, intraperiplasmic predators include *Bdellovibrio bacteriovorus*, *Halobacteriovorax* spp., *Peredibacter starrii*, *Bacteriovorax stolpii*, and *Pseudobacteriovorax antillogorgiicola* [23,40,41,42,43]. Among these, *Bdellovibrio bacteriovorus*, a Gram-negative bacterium with a single polar flagellum allowing for its high motility, is the most studied predator [20]. The life cycle of intraperiplasmic predators is complex. It has several stages that include attachment to the target bacterium, invasion, formation of a bdelloplast, elongation, septation via segmentation, and, eventually, lysis of the target bacterium and release of the progeny of the initial predatory bacterium [20,44,45]. Figure 1 shows the life cycle of intraperiplasmic predatory bacteria.

Despite their differences in the mechanistic way of action, a common feature of all predatory bacteria includes their ability to kill other bacteria and hydrolyze their macromolecules [20]. This is accomplished by the activity of several enzymes, such as nucleases and proteases encoded by their genomes [46,47,48,49]. Due to this activity, they have been evaluated for possible application in many different fields, such as biofilm removal [50,51], bioplastic recovery [52,53], and treatment of sludge [54,55]. Additionally, many studies focus on the use of predatory bacteria and, more commonly, of the *Bdellovibrio*- and–-like organisms (BALOs) as biocontrol agents, such as in the food industry [20,56,57].

Interestingly, prey evasion has been described and could become a theoretical barrier in the medical use of predatory bacteria. For example, *E. coli* has been extensively studied in pairs with *M. xanthus* due to their unique predator–prey relationship. Various ways of evading myxococcal invasion have been described, from biofilm formation to adaptations acquired in experimental co-evolution models [58,59]. Additionally, myxococcal predation exerts a genomic shift in experimental coculture models, showing a complex relationship [60]. Research by Zhang et al. also showed that mutants with deleted genes regulating flagella production contributed to anti-myxococcal resistance. Interestingly, when another gene was removed (dusB) in *E. coli*-reduced production of myxovirescin A, an antibiotic produced by *M. xanthus* was noted [61].

## 3. Non-Medical Applications of Predatory Bacteria

### 3.1. Food Industry

Microbial contamination can occur at various stages during food processing, such as production, processing, or distribution. This can cause a significant decline in productivity and threaten public health due to an increased likelihood of spreading pathogens associated with food poisoning and gastrointestinal disease [20]. Thus, preventing bacterial contamination and spread requires effective methods with broad activity against pathogens and activity against biofilms while being safe and compatible with foods. Several techniques for controlling food contamination are currently used, including heat and ultraviolet light, even though they all have some drawbacks that may limit their use, such as toxicity. At the same time, they may not effectively eliminate biofilms or may alter food quality [20,62,63].

The diversity of possible food-associated pathogens requires that treatments used to reduce the likelihood of bacterial contamination have a broad-spectrum capacity [20]. Moreover, these treatments would preferably have activity against biofilms, since some food pathogens could form biofilms when contaminating food. For example, *Escherichia coli* O157:H7 and *Salmonella* can cause food poisoning by growing on the surface of the meat and exposed equipment, forming biofilms, and then further spreading to other foods [64,65,66,67,68]. To that end, predatory bacteria could be used in the food industry, with *B. bacteriovorus* being the most well studied [20]. For example, one predatory strain, *B. bacteriovorus* 109J, was found to be able to significantly reduce the viability of several bacteria that can contaminate food, such as *Escherichia*, *Salmonella*, *Enterobacter*, *Shigella*, *Vibrio*, *Citrobacter*, and *Yersinia,* among others [69,70]. Similarly, another predatory bacterium, *M. aeruginosavorus*, also elicits broad-spectrum activity against several bacterial species and can significantly reduce the concentrations of *Escherichia*, *Shigella*, *Enterobacter*, *Yersinia*, and *Citrobacter* species [70]. However, some commonly studied bacterial predators, such as those mentioned above, have the disadvantage that they cannot prey on Gram-positive strains such as *Enterococcus* and *Staphylococcus* species [69,71]. Some other predatory bacteria, members of the wolf-pack predators, can attach to and consume Gram-positive bacteria, such as *Myxococcus* sp. MH1 [72].

Notably, a significant proportion of bacteria lives in biofilms, even in the food industry, on the surface of food, in workshops, and elsewhere [73,74]. These biofilms may resist killing with chemicals or antimicrobials, but they could be susceptible to predatory bacteria [20]. For example, Kadouri et al. have shown that different predatory bacteria were able to significantly reduce bacterial populations of *E. coli*, *Pseudomonas fluorescens*, *K. pneumoniae*, and *Pseudomonas aeruginosa* biofilms using *M. aeruginosavorus* and *B. bacteriovorus* 109J [75,76]. Ever since, several other groups have also shown that bacterial predators were able to prey on bacterial biofilms, as is the case with BALOs that were found to be able to dismantle *Salmonella enterica* biofilms [77,78]. More examples of the effect of predatory bacteria on biofilms and the possible applications in the food industry are reviewed separately [20].

Further on, predatory bacteria were shown to be safe, compatible with food, and with low immunogenic potential, thus posing a viable option for use in food safety [20]. More specifically, predatory bacteria are known in different sets of experiments to be unable to invade eukaryotic cells, and they only caused minimal cellular responses, such as cytokine production, even when the predatory bacteria were added at very high amounts [79,80]. Despite the above, predatory bacteria are also easy to control to increase safety after their application in the food industry. For example, simple detergents, such as those containing at least 0.02% sodium dodecyl sulfate (SDS), can cause complete or almost complete killing of predatory bacteria almost instantaneously. This implies that simple application of such a detergent followed by rinse can eliminate almost all the predators before consumption of foods such as fruits [18,81]. Another approach could include the radiation of food products, which has been shown to effectively kill predatory bacteria without harming butter lettuce when low doses of gamma irradiation were used [82,83].

### 3.2. Biocontrol

Beyond their potential for use in food safety, predatory bacteria could also be used as a biocontrol in the production of biofuel, as in the case of microalgae-derived biofuel [50]. More specifically, bacterial contamination in open ponds may affect microalgae growth. To that end, the use of *Bdellovibrio* may limit the contamination by other bacteria, thus allowing the growth of microalgae, leading to the production of green biofuel [84].

Additionally, there are reports of the use of predatory bacteria in agriculture [85]. For example, predatory bacteria have been used against phytopathogens, such as in the case of soybean blight that is caused by *Pseudomonas savastanoi* pv. *glycinea* [86]. More specifically, *B. bacteriovorus* was shown to effectively prey on the soybean pathogen and inhibit infection of the plant [86]. In another example, *B. bacteriovorus* was shown to protect against block soft rot disease in potatoes, and this effect was more evident when predatory bacteria were added before the pathogen [87]. Moreover, a strain of *Myxococcus xanthus* was effective in the control of tomato bacterial wilt that is caused by *Ralstonia solanacearum* [88].

In aquaculture, bacterial contamination is more commonly caused by *Vibrio* spp. οr *Aeromonas* spp. and can lead to disease outbreaks [85,89]. Using non-antibiotic methods to decrease bacterial contamination can be associated with significant benefits, since it could be associated with decreased exposure of the aquatic ecosystem to antimicrobials, thus reducing antimicrobial pollution [90]. For example, *Halobacteriovorax*, a marine group of BALOs, can prey on pathogenic strains of *Vibrio parahaemolyticus*, thus being able to promote the safety of seafood [91]. Furthermore, in vivo studies recently showed that *Halobacteriovorax* can also effectively reduce *Vibrio* species in mussels that can cause life-threatening infections [92]. In another example, the same predatory bacteria were found to significantly reduce the bacterial load of *Vibrio* in the hemolymph of lobsters without reducing host survival [93]. However, since the concept of the use of predatory bacteria in aquaculture is novel, further studies are needed to optimize the way they could be administered and determine their efficacy and safety [85].

Wastewater treatment plants are essential engineering ecosystems for reducing environmental pollution and protecting public health [85]. Several microbial processes are taking place in these plants, leading to the degradation of unwanted organic matter and the removal of phosphorus and nitrogen [94]. The use of predatory bacteria in wastewater treatment plants could lead to a reduction in the bacterial load of drug-resistant pathogens and significant activity on biofilms, leading to bacterial killing by the predatory bacteria themselves or by increased activity of the chemicals that are present in the plants [95].

**Figure 2:**
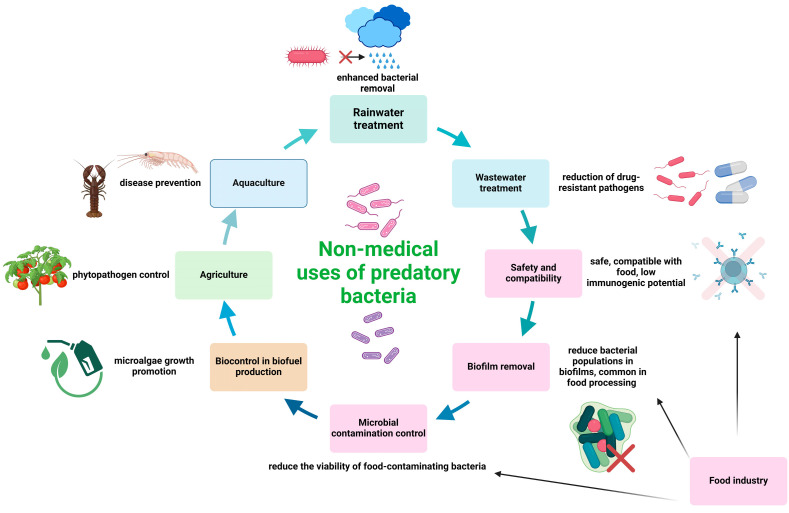
Non-medical applications of predatory bacteria. The image was created with Biorender.

Interestingly, predatory bacteria have also been studied as a disinfectant measure for rainwater treatment. To that end, predatory bacteria were found to efficiently enhance the removal of Gram-negative bacteria when applied as a pre-treatment to solar disinfection and photocatalysis [96]. Figure 2 summarizes the non-medical applications of predatory bacteria.

## 4. Medical Applications of Predatory Bacteria

Using predatory bacteria in clinical therapeutics requires adequate knowledge of their microbial target spectrum, properties, and potential adverse effects on the host organism regarding immune activation, toxicity, tissue damage, and the possibility of bacterial persistence [97]. Thus, in vitro and in vivo studies are a prerequisite to reaching the mature stage of clinical studies.

### 4.1. In Vitro Studies

Several in vitro studies have been conducted to examine the effect of predatory bacteria on their target bacteria. Additionally, in vitro studies have been conducted to examine the effect and safety of predatory bacteria in cell lines in tissue culture conditions [97]. Several such studies in human cell lines, such as with corneal-limbal epithelial cells, macrophages, monocytes, liver epithelial cells, kidney epithelial cells, and spleen monocytes, have been conducted with different numbers of predatory bacteria and for different periods, and pro- and anti-inflammatory cytokines were measured [79,80,98,99,100]. According to the results of these studies, even though the presence of predatory bacteria does induce the production of cytokines, their levels are very low, implying a negligible immunostimulatory potential by predatory bacteria per se [98,99,100]. This may be associated with their unique lipid A structure and the presence of a sheathed flagellum [97,98,101]. Other examples of encouraging in vitro studies include cytotoxicity measurements, cell viability imaging, and assessment of morphological changes performed with animal and human cell culture lines exposed to *B. bacteriovorus* that did not show evidence of toxicity in human cells [79,80,99]. In additional experiments, the uptake, persistence, and clearance of live predatory bacteria were assessed in human macrophage cell lines (U937) [100]. Raghunathan et al. used fluorescently labeled *B. bacteriovorus* to visualize the bacteria under the microscope and count live intracellular bacteria, and in another set of experiments, predatory bacteria that had been previously engulfed by the U937 macrophages were recovered and counted after previous experimental lysis of the macrophages. Even though the assessment of *B. bacteriovorus* numbers after their interaction with the macrophage cell lines is demanding, these experiments showed that a significant number of the predatory bacteria could survive for up to 24 h inside the macrophage cell lines, thus implying that the predatory bacteria could prey for a long time after their engulfment from the macrophages in the infected tissues [97]. Moreover, using specific inhibitors during the interaction of predatory bacteria with macrophage cell lines, a role for the actin cytoskeleton of the host was shown in the uptake of *B. bacteriovorus*, leading to trafficking through the phagolysosomal pathway [100]. Finally, the viability of eukaryotic cells was not affected [100]. In a different set of experiments, *B. bacteriovorus* was also found to survive phagocytosis and persist in murine macrophages for many hours in vitro, thus protecting SKH-1 mice from the lethal challenge of systemic plague [102].

Regarding in vitro experiments and the efficacy of predatory bacteria against clinical isolates, several studies have shown their potential. For example, Iebba et al. evaluated the predatory behavior of *B. bacteriovorus* against *P. aeruginosa* and *S. aureus* cystic fibrosis isolates with broth culture, static biofilms, field emission scanning electron microscope, flow biofilms, and a zymographic technique [103]. This study suggested that *B. bacteriovorus* could act as a living antibiotic in cystic fibrosis through a dual foraging system against Gram-positive (epibiotic) and Gram-negative (periplasmic) bacteria [103]. In a more recent study, Kahraman Vatansever et al. evaluated the effect of *B. bacteriovorus* HD100 on several clinical pathogens and their biofilms [104]. *B. bacteriovorus* was effective against Gram-negative isolates such as Enterobacterales, *Salmonella*, and *Stenotrophomonas.* Still, the activity against *P. aeruginosa* and *A. baumannii* was the lowest among Gram-negative bacteria. Interestingly, *B. bacteriovorus Staphylococcus* species in this study were also inhibited in co-culture studies, even though *B. bacteriovorus* was previously considered not able to prey on Gram-positive isolates [104]. *B. bacteriovorus* was also found in other in vitro studies to be able to prey against oral pathogens and periodontopathogens [70,105]. Importantly, in a relatively recent vitro study, *B. bacteriovorhus* and *M. aeruginosavorus* were found to be able to prey on colistin-resistant Gram-negative bacteria expressing mcr-1, such as *A. baumannii*, *E. coli*, *K. pneumoniae*, and *P. aeruginosa* [106]. Other MDR human clinical isolates have been evaluated and were found to be susceptible to *B. bacteriovorus* [99,107,108,109,110]. Additionally, *B. bacteriovorus* can reduce bacterial load in different settings, such as in laboratory buffer and human serum, and against target bacteria in biofilms [70,75,108,109].

### 4.2. In Vivo Studies

In terms of safety for use in animals, several sets of in vivo experiments have evaluated the viability of *Bdellovibrio* species in the intestines of vertebrates, either exothermic or endothermic [97]. For example, *B. bacteriovorus* was administered in the intestines of leopard frogs, catfish, rabbits, and mice but showed minimal or no recovery days after inoculation, and no pathogenicity associated with the predatory bacteria was reported [111]. In another study, *B. bacteriovorus* was also non-pathogenic when provided to chicken [112]. Moreover, *B. bacteriovorus* was non-immunogenic and non-toxic when deployed on the ocular surface of the eyes of rabbits [113]. Similarly, further experiments in mice, rats, and zebrafish larvae, using assessments of morbidity, production of pro- and anti-inflammatory cytokines, histopathology, bacterial growth, and other adverse events, proved that predatory bacteria are not harmful when given to these experimental models in vivo [102,114,115,116,117,118].

In terms of efficacy, *B. bacteriovorus* HD100 was shown to significantly reduce the bacterial load of *Salmonella* in the feces of chickens that had been previously experimentally infected with Salmonella enteritidis p125109. Furthermore, the cecum of chickens treated with the predatory bacteria had significantly fewer findings of inflammation compared to the control chickens that had not been treated with *B. bacteriovorus* [112]. Notably, *B. bacteriovorus* was not isolated from the cecum of treated chickens at the end of the trial, implying that these predatory bacteria had a short life in the intestines of the treated animals [112].

In a rat model, *B. bacteriovorus* 109J was administered intranasally to treat the subjects for *Klebsiella pneumoniae* respiratory tract infection and led to a significant reduction in bacterial burden in the treated animals compared to the control animals [114]. *B. bacteriovorus* 109J was also evaluated in a model of experimental bacteremia. More specifically, rats were treated with *K. pneumoniae* in their tail veins and were then intravenously treated with *B. bacteriovorus* 109J [115]. Treatment with predatory bacteria did not significantly reduce *K. pneumoniae* burden in the blood and failed to prevent bacterial dissemination to other organs. Of note, predatory bacteria were efficiently cleared from the bloodstream of rats within 20 days after their injection [115].

In another set of experiments using zebrafish, which allow for innovative live microscopy due to their transparent nature, homology to humans, and well-studied immune system, predatory bacteria were studied in a localized larval infection model [97,118,119,120,121,122]. In an interesting study, Willis et al. characterized the in vivo predation of a fluorescently GFP-labeled *Shigella flexneri* by fluorescently mCherry-labeled *B. bacteriovorus* over time after infection of zebrafish larvae using live-cell imaging [118]. In the control larvae that had been infected by *S. flexneri* but were not treated with *B. bacteriovorus*, an increasing bacterial burden was noted, as shown by the increasing GFP fluorescence. Larvae treated with *B. bacteriovorus* showed a significant reduction in GFP fluorescence. Enumeration of both bacterial species and confocal microscopy that showed more evidence regarding live bacterial predation inside the larvae further supported the above findings [118].

Moreover, in a relatively recent study, Russo et al. found that *B. bacteriovorus* 109J significantly reduced the bacterial load of *Yersinia pestis* in the lungs of mice infected experimentally [123]. In another study, SKH-1 mice pre-treated with *B. bacteriovorus* HD100 via intraperitoneal injection were infected with a lethal dose of *Y. pestis* CO92 and were then treated daily with *B. bacteriovorus*, and the bacterial spread of *Y. pestis* was observed [102]. Treatment with *B. bacteriovorus* was associated with significantly lower bacterial numbers of *Y. pestis*, as assessed by luciferase signal and splenic bacterial load counts at the end of the experiment. However, no similar protection was noted for Balb/c mice infected with *Y. pestis* and then treated with *B. bacteriovorus* in another set of experiments, implying that the protective effect by the predatory bacteria could depend on the immunologic or genetic background of the animal [102].

On the other hand, when calves that had been experimentally infected with *Moraxella bovis*, a microbial cause of infectious bovine keratoconjunctivitis, were treated with *B. bacteriovorus* 109J, no significant improvement of corneal ulcer formation was noted compared to the untreated calves [124]. This is contrary to the previous observation that *B. bacteriovorus* could effectively control *M. bovis* in a tissue culture model in vitro [125].

In a more recent study, Romanowski et al. evaluated the ability of *B. bacteriovorus* and *M. aeruginosavorus* to limit the intraocular growth of *S. aureus*, *P. aeruginosa*, and *Serratia marcescens* in a New Zealand white rabbit endophthalmitis model [126]. Even though these predatory bacteria could not significantly inhibit the growth of *S. aureus*, they could reduce the growth of *P. aeruginosa* and, to a smaller extent, of *S. marcescens*.

Due to the relatively little experience on the topic and the sometimes contradictory results of the in vivo studies of predatory bacteria, no clinical trial exists until now in humans. However, the need for alternative treatments for infectious diseases in humans, especially in the era of AMR, and the presence of mounting evidence for the safety and the relative efficacy of predatory bacteria treatment in animals, as shown by the abovementioned positive findings, gives promise for further testing of predatory bacteria even in clinical trials in humans [97].

### 4.3. Predatory Bacteria Metabolites as Antimicrobials

A bioinformatics analysis of the genome of *B. bacteriovorus* HD100 revealed about 193 potential lytic proteins, including 150 peptidases and proteases, 20 DNases, 9 RNases, and other enzymes [127]. This has led to the possibility of using these predatory bacteria’s products in several biotechnological processes [50,127]. In a study by Monnappa et al., the extracellular enzymes produced by a host-independent *B. bacteriovorus* strain were assessed against biofilms of *S. aureus* and were effective [128]. More specifically, the supernatant from host-independent *B. bacteriovorus* was added in the wells of plates containing biofilms of *S. aureus*, and fluorescent and scanning electron microscopy confirmed significant disruption of the biofilms after 24 h of exposure to the supernatant [128]. This was also associated with a fivefold reduction in the infectivity of the staphylococci. Examination of the content of the supernatant led to the identification of specific proteases and DNases, and it was hypothesized that these enzymes reduced the virulence of staphylococci, thus implying that enzymes derived from predatory bacteria could act as antimicrobials [129]. Moreover, *Archangium lipolyticum* sp., recently discovered in a pig farm’s soil, exhibits predation and efficient destruction via lipolysis of resistant microorganisms, such as *E. coli* 64 and MRSA GDMCC 1.771. The bacteriolytic properties of this novel myxobacterium were attributed, after genomic analysis and enzymatic extraction, to a lipase ArEstA. In in vitro conditions, it exhibited bacteriolytic activity versus *E. coli* 64 but not versus the aforementioned MRSA strain, probably due to enzymatic accessibility issues to lipid substrates [130].

### 4.4. Combining Treatment with Predatory Bacteria

Due to the increasing problem of AMR, other approaches involving non-antimicrobial options have been evaluated [12,13,14]. In some cases, monotherapy with such modalities may be associated with effective treatment; however, combination treatment could be associated, at least theoretically, with a higher efficacy [129]. For example, predatory bacteria and bacteriophages can be considered to have a relatively similar mechanism of action, since they are self-replicating and self-limiting, being able to multiply only within specifically susceptible host bacteria. Moreover, they are both considered to have relatively few adverse effects [97]. Combining predatory bacteria such as *Bdellovibrio* species with bacteriophages or antimicrobials or the use of enzymes derived from predatory bacteria have been considered as alternative treatment modalities [50,57]. For example, Hobley et al. used a double predation model of a rosette-tailed-like bacteriophage and *B. bacteriovorus* HD100 against *E. coli* [131]. They identified a very high efficacy, almost completely eradicating the prey bacteria in liquid culture.

In another study by Im et al., the efficacy of combination treatment with *B. bacteriovorus* HD100 and violacein (an antimicrobial agent specifically targeting Gram-positive microorganisms) against six different Gram-positive and Gram-negative bacteria (*S. aureus*, *Bacillus cereus*, *Staphylococcus epidermis*, *E. coli*, *K. pneumoniae*, and *Acinetobacter baumannii*) was examined [110]. Combination treatment with *B. bacteriovorus* HD100 and violacein in polymicrobial cultures led to a significant reduction in bacterial load, thus implying the presence of synergy among the predatory bacteria and violacein.

Of note, co-administration of predatory bacteria with antimicrobials would necessitate careful selection of the antimicrobial, since the predatory bacteria that would be used should be resistant to the antimicrobial [129]. However, antimicrobial susceptibility to predatory bacteria may be challenging given their dependency on prey bacteria for proliferation, and no specific breakpoints for antimicrobial resistance exist until now [81]. To that end, there are attempts to develop assays allowing for the evaluation of antimicrobial susceptibility of predatory bacteria in liquid-based assays with prey bacteria in a stationary phase in a nutrient-limited medium with different concentrations of the antimicrobials to be tested [132]. Among the currently used antimicrobials, trimethoprim shows the lowest antimicrobial activity against *B. bacteriovorus* and could be used in combination with that predatory bacterium [129].

**Figure 3:**
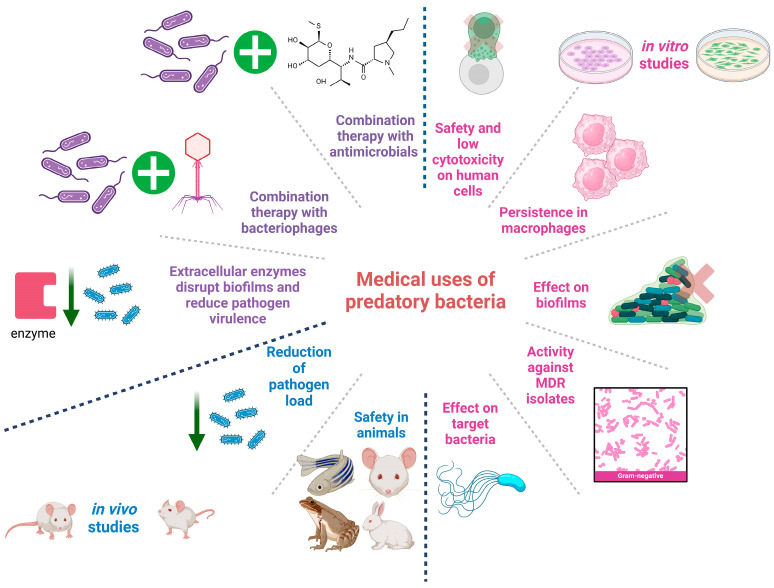
Medical applications of predatory bacteria. The image was created with Biorender.

Lastly, combination treatment with different predatory bacteria could be used in the future; however, no studies supporting this concept were identified during the preparation of this manuscript. Figure 3 summarizes the potential medical applications of predatory bacteria.

## 5. Conclusions and Future Remarks

Given the increasing AMR and the need for novel non-antimicrobial treatments, the potential for using predatory bacteria in humans would allow for treating bacteria with limited treatment options. Given their promising results in terms of safety and efficacy in in vitro and in vivo experiments, future studies in human clinical trials would allow for appropriately evaluating these bacteria for the treatment of bacterial infections either alone or in combination with classic antimicrobials or other novel modalities, such as bacteriophages.

## Data Availability

The data presented in this study are available on request from the corresponding author.

## References

[1] Abraham E.P., Chain E. (1988). An Enzyme from Bacteria Able to Destroy Penicillin. 1940. Rev. Infect. Dis..

[2] Lobanovska M., Pilla G. (2017). Penicillin’s Discovery and Antibiotic Resistance: Lessons for the Future?. Yale J. Biol. Med..

[3] Spellberg B., Guidos R., Gilbert D., Bradley J., Boucher H.W., Scheld W.M., Bartlett J.G., Edwards J., Infectious Diseases Society of America (2008). The Epidemic of Antibiotic-Resistant Infections: A Call to Action for the Medical Community from the Infectious Diseases Society of America. Clin. Infect. Dis. Off. Publ. Infect. Dis. Soc. Am..

[4] Magiorakos A.-P., Srinivasan A., Carey R.B., Carmeli Y., Falagas M.E., Giske C.G., Harbarth S., Hindler J.F., Kahlmeter G., Olsson-Liljequist B. (2012). Multidrug-Resistant, Extensively Drug-Resistant and Pandrug-Resistant Bacteria: An International Expert Proposal for Interim Standard Definitions for Acquired Resistance. Clin. Microbiol. Infect. Off. Publ. Eur. Soc. Clin. Microbiol. Infect. Dis..

[5] Hou J., Long X., Wang X., Li L., Mao D., Luo Y., Ren H. (2023). Global Trend of Antimicrobial Resistance in Common Bacterial Pathogens in Response to Antibiotic Consumption. J. Hazard. Mater..

[6] Karakonstantis S., Kritsotakis E.I., Gikas A. (2020). Pandrug-Resistant Gram-Negative Bacteria: A Systematic Review of Current Epidemiology, Prognosis and Treatment Options. J. Antimicrob. Chemother..

[7] Falagas M.E., Kasiakou S.K. (2005). Colistin: The Revival of Polymyxins for the Management of Multidrug-Resistant Gram-Negative Bacterial Infections. Clin. Infect. Dis. Off. Publ. Infect. Dis. Soc. Am..

[8] Samson I. (2005). A New Class of Antimycobacterial Drugs: The Diarylquinolines. Thorax.

[9] Ling L.L., Schneider T., Peoples A.J., Spoering A.L., Engels I., Conlon B.P., Mueller A., Schäberle T.F., Hughes D.E., Epstein S. (2015). A New Antibiotic Kills Pathogens without Detectable Resistance. Nature.

[10] Hover B.M., Kim S.-H., Katz M., Charlop-Powers Z., Owen J.G., Ternei M.A., Maniko J., Estrela A.B., Molina H., Park S. (2018). Culture-Independent Discovery of the Malacidins as Calcium-Dependent Antibiotics with Activity against Multidrug-Resistant Gram-Positive Pathogens. Nat. Microbiol..

[11] Butler M.S., Henderson I.R., Capon R.J., Blaskovich M.A.T. (2023). Antibiotics in the Clinical Pipeline as of December 2022. J. Antibiot..

[12] Ioannou P., Baliou S., Samonis G. (2023). Bacteriophages in Infectious Diseases and Beyond—A Narrative Review. Antibiotics.

[13] Ioannou P., Baliou S., Kofteridis D.P. (2023). Antimicrobial Peptides in Infectious Diseases and Beyond-A Narrative Review. Life.

[14] Ioannou P., Baliou S., Samonis G. (2024). Nanotechnology in the Diagnosis and Treatment of Antibiotic-Resistant Infections. Antibiotics.

[15] Hungate B.A., Marks J.C., Power M.E., Schwartz E., van Groenigen K.J., Blazewicz S.J., Chuckran P., Dijkstra P., Finley B.K., Firestone M.K. (2021). The Functional Significance of Bacterial Predators. mBio.

[16] Makowski Ł., Trojanowski D., Till R., Lambert C., Lowry R., Sockett R.E., Zakrzewska-Czerwińska J. (2019). Dynamics of Chromosome Replication and Its Relationship to Predatory Attack Lifestyles in *Bdellovibrio bacteriovorus*. Appl. Environ. Microbiol..

[17] Cohen Y., Pasternak Z., Müller S., Hübschmann T., Schattenberg F., Sivakala K.K., Abed-Rabbo A., Chatzinotas A., Jurkevitch E. (2021). Community and Single Cell Analyses Reveal Complex Predatory Interactions between Bacteria in High Diversity Systems. Nat. Commun..

[18] Jang H., Mun W., Choi S.Y., Mitchell R.J. (2022). Use of Resazurin to Rapidly Enumerate *Bdellovibrio* and like Organisms and Evaluate Their Activities. Microbiol. Spectr..

[19] Pineiro S., Chauhan A., Berhane T., Athar R., Zheng G., Wang C., Dickerson T., Liang X., Lymperopoulou D.S., Chen H. (2013). Niche Partition of *Bacteriovorax* Operational Taxonomic Units along Salinity and Temporal Gradients in the Chesapeake Bay Reveals Distinct Estuarine Strains. Microb. Ecol..

[20] Mun W., Choi S.Y., Upatissa S., Mitchell R.J. (2023). Predatory Bacteria as Potential Biofilm Control and Eradication Agents in the Food Industry. Food Sci. Biotechnol..

[21] Jurkevitch E., Minz D., Ramati B., Barel G. (2000). Prey Range Characterization, Ribotyping, and Diversity of Soil and Rhizosphere *Bdellovibrio* spp. Isolated on Phytopathogenic Bacteria. Appl. Environ. Microbiol..

[22] Oyedara O.O., De Luna-Santillana E.D.J., Olguin-Rodriguez O., Guo X., Mendoza-Villa M.A., Menchaca-Arredondo J.L., Elufisan T.O., Garza-Hernandez J.A., Garcia Leon I., Rodriguez-Perez M.A. (2016). Isolation of *Bdellovibrio* sp. from Soil Samples in Mexico and Their Potential Applications in Control of Pathogens. MicrobiologyOpen.

[23] Baer M.L., Ravel J., Piñeiro S.A., Guether-Borg D., Williams H.N. (2004). Reclassification of Salt-Water *Bdellovibrio* sp. as *Bacteriovorax marinus* sp. Nov. and *Bacteriovorax litoralis* sp. Nov. Int. J. Syst. Evol. Microbiol..

[24] Mun W., Upatissa S., Lim S., Dwidar M., Mitchell R.J. (2022). Outer Membrane Porin F in *E. coli* Is Critical for Effective Predation by *Bdellovibrio*. Microbiol. Spectr..

[25] Guo Y., Pan Q., Yan S., Chen Y., Li M., Chen D., Han H., Wu B., Cai J. (2017). *Bdellovibrio* and like Organisms Promoted Growth and Survival of Juvenile Abalone Haliotis Discus Hannai Ino and Modulated Bacterial Community Structures in Its Gut. Aquac. Int..

[26] Schwudke D., Strauch E., Krueger M., Appel B. (2001). Taxonomic Studies of Predatory *Bdellovibrios* Based on 16S rRNA Analysis, Ribotyping and the Hit Locus and Characterization of Isolates from the Gut of Animals. Syst. Appl. Microbiol..

[27] Seccareccia I., Kost C., Nett M. (2015). Quantitative Analysis of Lysobacter Predation. Appl. Environ. Microbiol..

[28] Livingstone P.G., Morphew R.M., Whitworth D.E. (2017). Myxobacteria Are Able to Prey Broadly upon Clinically-Relevant Pathogens, Exhibiting a Prey Range Which Cannot Be Explained by Phylogeny. Front. Microbiol..

[29] Xiao Y., Wei X., Ebright R., Wall D. (2011). Antibiotic Production by Myxobacteria Plays a Role in Predation. J. Bacteriol..

[30] Evans A.G.L., Davey H.M., Cookson A., Currinn H., Cooke-Fox G., Stanczyk P.J., Whitworth D.E. (2012). Predatory Activity of *Myxococcus xanthus* Outer-Membrane Vesicles and Properties of Their Hydrolase Cargo. Microbiology.

[31] McBride M.J., Zusman D.R. (1996). Behavioral Analysis of Single Cells of *Myxococcus xanthus* in Response to Prey Cells of *Escherichia coli*. FEMS Microbiol. Lett..

[32] Berleman J.E., Kirby J.R. (2009). Deciphering the Hunting Strategy of a Bacterial Wolfpack. FEMS Microbiol. Rev..

[33] Muñoz-Dorado J., Marcos-Torres F.J., García-Bravo E., Moraleda-Muñoz A., Pérez J. (2016). Myxobacteria: Moving, Killing, Feeding, and Surviving Together. Front. Microbiol..

[34] Shimkets L.J. (1999). Intercellular Signaling during Fruiting-Body Development of *Myxococcus xanthus*. Annu. Rev. Microbiol..

[35] Guerrero R., Pedros-Alio C., Esteve I., Mas J., Chase D., Margulis L. (1986). Predatory Prokaryotes: Predation and Primary Consumption Evolved in Bacteria. Proc. Natl. Acad. Sci. USA.

[36] Koval S.F., Hynes S.H., Flannagan R.S., Pasternak Z., Davidov Y., Jurkevitch E. (2013). *Bdellovibrio exovorus* sp. Nov., a Novel Predator of *Caulobacter crescentus*. Int. J. Syst. Evol. Microbiol..

[37] Pasternak Z., Njagi M., Shani Y., Chanyi R., Rotem O., Lurie-Weinberger M.N., Koval S., Pietrokovski S., Gophna U., Jurkevitch E. (2014). In and out: An Analysis of Epibiotic vs Periplasmic Bacterial Predators. ISME J..

[38] Hovde B.T., Steichen S.A., Starkenburg S.R., Brown J.K. (2020). *Vampirovibrio chlorellavorus* Draft Genome Sequence, Annotation, and Preliminary Characterization of Pathogenicity Determinants. Phycol. Res..

[39] Moreira D., Zivanovic Y., López-Archilla A.I., Iniesto M., López-García P. (2021). Reductive Evolution and Unique Predatory Mode in the CPR Bacterium *Vampirococcus lugosii*. Nat. Commun..

[40] Seideler R.J., Mandel M., Baptist J.N. (1972). Molecular Heterogeneity of the *Bdellovibrios*: Evidence of Two New Species. J. Bacteriol..

[41] Im H., Kwon H., Cho G., Kwon J., Choi S.Y., Mitchell R.J. (2019). Viscosity Has Dichotomous Effects on *Bdellovibrio bacteriovorus* HD100 Predation. Environ. Microbiol..

[42] Sathyamoorthy R., Maoz A., Pasternak Z., Im H., Huppert A., Kadouri D., Jurkevitch E. (2019). Bacterial Predation under Changing Viscosities. Environ. Microbiol..

[43] McCauley E.P., Haltli B., Kerr R.G. (2015). Description of *Pseudobacteriovorax antillogorgiicola* Gen. Nov., sp. Nov., a Bacterium Isolated from the Gorgonian Octocoral *Antillogorgia elisabethae*, Belonging to the Family *Pseudobacteriovoracaceae* Fam. Nov., within the Order *Bdellovibrionales*. Int. J. Syst. Evol. Microbiol..

[44] Fenton A.K., Kanna M., Woods R.D., Aizawa S.-I., Sockett R.E. (2010). Shadowing the Actions of a Predator: Backlit Fluorescent Microscopy Reveals Synchronous Nonbinary Septation of Predatory *Bdellovibrio* inside Prey and Exit through Discrete Bdelloplast Pores. J. Bacteriol..

[45] Rotem O., Pasternak Z., Shimoni E., Belausov E., Porat Z., Pietrokovski S., Jurkevitch E. (2015). Cell-Cycle Progress in Obligate Predatory Bacteria Is Dependent upon Sequential Sensing of Prey Recognition and Prey Quality Cues. Proc. Natl. Acad. Sci. USA.

[46] Oyedara O.O., Segura-Cabrera A., Guo X., Elufisan T.O., Cantú González R.A., Rodríguez Pérez M.A. (2018). Whole-Genome Sequencing and Comparative Genome Analysis Provided Insight into the Predatory Features and Genetic Diversity of Two *Bdellovibrio* Species Isolated from Soil. Int. J. Genom..

[47] Inoue D., Hiroshima N., Ishizawa H., Dohra H., Ike M. (2022). Complete Genome Sequences of Two Predatory Bacterial Strains, *Bacteriovorax* sp. HI3 and *Myxococcus* sp. MH1, Isolated from a Freshwater Pond. Microbiol. Resour. Announc..

[48] Pasternak Z., Pietrokovski S., Rotem O., Gophna U., Lurie-Weinberger M.N., Jurkevitch E. (2013). By Their Genes Ye Shall Know Them: Genomic Signatures of Predatory Bacteria. ISME J..

[49] Williams L.E., Cullen N., DeGiorgis J.A., Martinez K.J., Mellone J., Oser M., Wang J., Zhang Y. (2019). Variation in Genome Content and Predatory Phenotypes between *Bdellovibrio* sp. NC01 Isolated from Soil and *B. bacteriovorus* Type Strain HD100. Microbiology.

[50] Bratanis E., Andersson T., Lood R., Bukowska-Faniband E. (2020). Biotechnological Potential of *Bdellovibrio* and like Organisms and Their Secreted Enzymes. Front. Microbiol..

[51] Dwidar M., Monnappa A.K., Mitchell R.J. (2012). The Dual Probiotic and Antibiotic Nature of *Bdellovibrio bacteriovorus*. BMB Rep..

[52] Martínez V., Herencias C., Jurkevitch E., Prieto M.A. (2016). Engineering a Predatory Bacterium as a Proficient Killer Agent for Intracellular Bio-Products Recovery: The Case of the Polyhydroxyalkanoates. Sci. Rep..

[53] Martínez V., Jurkevitch E., García J.L., Prieto M.A. (2013). Reward for *Bdellovibrio bacteriovorus* for Preying on a Polyhydroxyalkanoate Producer. Environ. Microbiol..

[54] Feng S., Tan C.H., Constancias F., Kohli G.S., Cohen Y., Rice S.A. (2017). Predation by *Bdellovibrio bacteriovorus* Significantly Reduces Viability and Alters the Microbial Community Composition of Activated Sludge Flocs and Granules. FEMS Microbiol. Ecol..

[55] Yan C., Zhan M., Xv K., Zhang S., Liang T., Yu R. (2022). Sludge Dewaterability Enhancement under Low Temperature Condition with Cold-Tolerant *Bdellovibrio* sp. CLL13. Sci. Total Environ..

[56] Choi S.Y., Im H., Mitchell R.J. (2017). Violacein and Bacterial Predation: Promising Alternatives for Priority Multidrug Resistant Human Pathogens. Future Microbiol..

[57] Pérez J., Contreras-Moreno F.J., Marcos-Torres F.J., Moraleda-Muñoz A., Muñoz-Dorado J. (2020). The Antibiotic Crisis: How Bacterial Predators Can Help. Comput. Struct. Biotechnol. J..

[58] DePas W.H., Syed A.K., Sifuentes M., Lee J.S., Warshaw D., Saggar V., Csankovszki G., Boles B.R., Chapman M.R. (2014). Biofilm Formation Protects *Escherichia coli* against Killing by *Caenorhabditis elegans* and *Myxococcus xanthus*. Appl. Environ. Microbiol..

[59] Nair R.R., Vasse M., Wielgoss S., Sun L., Yu Y.-T.N., Velicer G.J. (2019). Bacterial Predator-Prey Coevolution Accelerates Genome Evolution and Selects on Virulence-Associated Prey Defences. Nat. Commun..

[60] Livingstone P.G., Millard A.D., Swain M.T., Whitworth D.E. (2018). Transcriptional Changes When *Myxococcus xanthus* Preys on *Escherichia coli* Suggest Myxobacterial Predators Are Constitutively Toxic but Regulate Their Feeding. Microb. Genom..

[61] Zhang N., Li T., Pan H., Wang Y., Li Q., Luan J., He X., Shi W., Li Y., Wang C. (2023). Genetic Components of *Escherichia coli* Involved in Its Complex Prey-Predator Interaction with *Myxococcus xanthus*. Front. Microbiol..

[62] Sert D., Mercan E., Kara Ü. (2020). Butter Production from Ozone-Treated Cream: Effects on Characteristics of Physicochemical, Microbiological, Thermal and Oxidative Stability. LWT.

[63] Baggio A., Marino M., Innocente N., Celotto M., Maifreni M. (2020). Antimicrobial Effect of Oxidative Technologies in Food Processing: An Overview. Eur. Food Res. Technol..

[64] Collineau L., Chapman B., Bao X., Sivapathasundaram B., Carson C.A., Fazil A., Reid-Smith R.J., Smith B.A. (2020). A Farm-to-Fork Quantitative Risk Assessment Model for Salmonella Heidelberg Resistant to Third-Generation Cephalosporins in Broiler Chickens in Canada. Int. J. Food Microbiol..

[65] Rothrock M.J., Guard J.Y., Oladeinde A. (2021). *Salmonella* Diversity along the Farm-to-Fork Continuum of Pastured Poultry Flocks in the Southeastern United States. Front. Anim. Sci..

[66] Dourou D., Beauchamp C.S., Yoon Y., Geornaras I., Belk K.E., Smith G.C., Nychas G.-J.E., Sofos J.N. (2011). Attachment and Biofilm Formation by *Escherichia coli* O157:H7 at Different Temperatures, on Various Food-Contact Surfaces Encountered in Beef Processing. Int. J. Food Microbiol..

[67] Silagyi K., Kim S.-H., Lo Y.M., Wei C. (2009). Production of Biofilm and Quorum Sensing by *Escherichia coli* O157:H7 and Its Transfer from Contact Surfaces to Meat, Poultry, Ready-to-Eat Deli, and Produce Products. Food Microbiol..

[68] Wang H., Ding S., Dong Y., Ye K., Xu X., Zhou G. (2013). Biofilm Formation of *Salmonella* Serotypes in Simulated Meat Processing Environments and Its Relationship to Cell Characteristics. J. Food Prot..

[69] Dashiff A., Junka R.A., Libera M., Kadouri D.E. (2011). Predation of Human Pathogens by the Predatory Bacteria *Micavibrio aeruginosavorus* and *Bdellovibrio bacteriovorus*. J. Appl. Microbiol..

[70] Dashiff A., Kadouri D.E. (2011). Predation of Oral Pathogens by *Bdellovibrio bacteriovorus* 109J. Mol. Oral Microbiol..

[71] Im H., Dwidar M., Mitchell R.J. (2018). *Bdellovibrio bacteriovorus* HD100, a Predator of Gram-Negative Bacteria, Benefits Energetically from *Staphylococcus aureus* Biofilms without Predation. ISME J..

[72] Inoue D., Hiroshima N., Nakamura S., Ishizawa H., Ike M. (2022). Characterization of Two Novel Predatory Bacteria, *Bacteriovorax Stolpii* HI3 and *Myxococcus* sp. MH1, Isolated from a Freshwater Pond: Prey Range, and Predatory Dynamics and Efficiency. Microorganisms.

[73] Flemming H.-C., Wuertz S. (2019). Bacteria and Archaea on Earth and Their Abundance in Biofilms. Nat. Rev. Microbiol..

[74] Galié S., García-Gutiérrez C., Miguélez E.M., Villar C.J., Lombó F. (2018). Biofilms in the Food Industry: Health Aspects and Control Methods. Front. Microbiol..

[75] Kadouri D., O’Toole G.A. (2005). Susceptibility of Biofilms to *Bdellovibrio bacteriovorus* Attack. Appl. Environ. Microbiol..

[76] Kadouri D., Venzon N.C., O’Toole G.A. (2007). Vulnerability of Pathogenic Biofilms to *Micavibrio aeruginosavorus*. Appl. Environ. Microbiol..

[77] Joseph B., Otta S.K., Karunasagar I., Karunasagar I. (2001). Biofilm Formation by *Salmonella* spp. on Food Contact Surfaces and Their Sensitivity to Sanitizers. Int. J. Food Microbiol..

[78] Merino L., Procura F., Trejo F.M., Bueno D.J., Golowczyc M.A. (2019). Biofilm Formation by *Salmonella* sp. in the Poultry Industry: Detection, Control and Eradication Strategies. Food Res. Int. Ott. Ont.

[79] Gupta S., Tang C., Tran M., Kadouri D.E. (2016). Effect of Predatory Bacteria on Human Cell Lines. PLoS ONE.

[80] Monnappa A.K., Bari W., Choi S.Y., Mitchell R.J. (2016). Investigating the Responses of Human Epithelial Cells to Predatory Bacteria. Sci. Rep..

[81] Cho G., Kwon J., Soh S.M., Jang H., Mitchell R.J. (2019). Sensitivity of Predatory Bacteria to Different Surfactants and Their Application to Check Bacterial Predation. Appl. Microbiol. Biotechnol..

[82] Niemira B.A., Fan X. (2014). Fruits and vegetables|Advances in Processing Technologies to Preserve and Enhance the Safety of Fresh and Fresh-Cut Fruits and Vegetables. Encyclopedia of Food Microbiology.

[83] Olanya O.M., Niemira B.A., Cassidy J.M., Boyd G., Uknalis J. (2020). Pathogen Reduction by Predatory Bacteria and Survival of *Bdellovibrio bacteriovorus* and *Escherichia coli* on Produce and Buffer Treated with Low-Dose Gamma Radiation. LWT.

[84] Li Y., Qiu F., Yan H., Wan X., Wang M., Ren K., Xu Q., Lv L., Yin C., Liu X. (2018). Increasing the Autotrophic Growth of *Chlorella* USTB-01 via the Control of Bacterial Contamination by *Bdellovibrio* USTB-06. J. Appl. Microbiol..

[85] Zhang L., Guo L., Cui Z., Ju F. (2024). Exploiting Predatory Bacteria as Biocontrol Agents across Ecosystems. Trends Microbiol..

[86] Scherff R.H. (1973). Control of Bacterial Blight of Soybean by *Bdellovibrio bacteriovorus*. Phytopathology.

[87] Youdkes D., Helman Y., Burdman S., Matan O., Jurkevitch E. (2020). Potential Control of Potato Soft Rot Disease by the Obligate Predators *Bdellovibrio* and like Organisms. Appl. Environ. Microbiol..

[88] Dong H., Xu X., Gao R., Li Y., Li A., Yao Q., Zhu H. (2021). *Myxococcus xanthus* R31 Suppresses Tomato Bacterial Wilt by Inhibiting the Pathogen *Ralstonia solanacearum* with Secreted Proteins. Front. Microbiol..

[89] Najnine F., Cao Q., Zhao Y., Cai J., Jurkevitch E., Mitchell R.J. (2020). Antibacterial Activities of *Bdellovibrio* and like Organisms in Aquaculture. The Ecology of Predation at the Microscale.

[90] Lyu J., Yang L., Zhang L., Ye B., Wang L. (2020). Antibiotics in Soil and Water in China—A Systematic Review and Source Analysis. Environ. Pollut..

[91] Richards G.P., Watson M.A., Williams H.N., Jones J.L. (2023). Predator-Prey Interactions between *Halobacteriovorax* and Pathogenic *Vibrio parahaemolyticus* Strains: Geographical Considerations and Influence of *Vibrio* Hemolysins. Microbiol. Spectr..

[92] Ottaviani D., Pieralisi S., Chierichetti S., Rocchegiani E., Hattab J., Mosca F., Tiscar P.G., Leoni F., Angelico G. (2020). *Vibrio parahaemolyticus* Control in Mussels by a *Halobacteriovorax* Isolated from the Adriatic Sea, Italy. Food Microbiol..

[93] Ooi M.C., Goulden E.F., Smith G.G., Bridle A.R. (2021). Predatory Bacteria in the Haemolymph of the Cultured Spiny Lobster *Panulirus ornatus*. Microbiology.

[94] Ju F., Xia Y., Guo F., Wang Z., Zhang T. (2014). Taxonomic Relatedness Shapes Bacterial Assembly in Activated Sludge of Globally Distributed Wastewater Treatment Plants. Environ. Microbiol..

[95] Mookherjee A., Jurkevitch E. (2022). Interactions between *Bdellovibrio* and like Organisms and Bacteria in Biofilms: Beyond Predator-Prey Dynamics. Environ. Microbiol..

[96] Waso M., Khan S., Singh A., McMichael S., Ahmed W., Fernández-Ibáñez P., Byrne J.A., Khan W. (2020). Predatory Bacteria in Combination with Solar Disinfection and Solar Photocatalysis for the Treatment of Rainwater. Water Res..

[97] Atterbury R.J., Tyson J. (2021). Predatory Bacteria as Living Antibiotics—Where Are We Now?. Microbiology.

[98] Schwudke D., Linscheid M., Strauch E., Appel B., Zahringer U., Moll H., Muller M., Brecker L., Gronow S., Lindner B. (2003). The Obligate Predatory *Bdellovibrio bacteriovorus* Possesses a Neutral Lipid A Containing Alpha-D-Mannoses That Replace Phosphate Residues: Similarities and Differences between the Lipid as and the Lipopolysaccharides of the Wild Type Strain B. bacteriovorus HD100 and Its Host-Independent Derivative HI100. J. Biol. Chem..

[99] Shanks R.M.Q., Davra V.R., Romanowski E.G., Brothers K.M., Stella N.A., Godboley D., Kadouri D.E. (2013). An Eye to a Kill: Using Predatory Bacteria to Control Gram-Negative Pathogens Associated with Ocular Infections. PLoS ONE.

[100] Raghunathan D., Radford P.M., Gell C., Negus D., Moore C., Till R., Tighe P.J., Wheatley S.P., Martinez-Pomares L., Sockett R.E. (2019). Engulfment, Persistence and Fate of *Bdellovibrio bacteriovorus* Predators inside Human Phagocytic Cells Informs Their Future Therapeutic Potential. Sci. Rep..

[101] Seidler R.J., Starr M.P. (1968). Structure of the Flagellum of *Bdellovibrio bacteriovorus*. J. Bacteriol..

[102] Findlay J.S., Flick-Smith H.C., Keyser E., Cooper I.A., Williamson E.D., Oyston P.C.F. (2019). Predatory Bacteria Can Protect SKH-1 Mice from a Lethal Plague Challenge. Sci. Rep..

[103] Iebba V., Totino V., Santangelo F., Gagliardi A., Ciotoli L., Virga A., Ambrosi C., Pompili M., De Biase R.V., Selan L. (2014). *Bdellovibrio bacteriovorus* Directly Attacks *Pseudomonas aeruginosa* and *Staphylococcus aureus* Cystic Fibrosis Isolates. Front. Microbiol..

[104] Kahraman Vatansever S., Tekintas Y., Cilli F.F., Hosgor-Limoncu M. (2023). Effect of Predator Bacteria *Bdellovibrio bacteriovorus* on Clinical Pathogens and Biofilms. Indian J. Microbiol..

[105] Patini R., Cattani P., Marchetti S., Isola G., Quaranta G., Gallenzi P. (2019). Evaluation of Predation Capability of Periodontopathogens Bacteria by *Bdellovibrio bacteriovorus* HD100. An in Vitro Study. Mater. Basel Switz..

[106] Dharani S., Kim D.H., Shanks R.M.Q., Doi Y., Kadouri D.E. (2018). Susceptibility of Colistin-Resistant Pathogens to Predatory Bacteria. Res. Microbiol..

[107] Kadouri D.E., To K., Shanks R.M.Q., Doi Y. (2013). Predatory Bacteria: A Potential Ally against Multidrug-Resistant Gram-Negative Pathogens. PLoS ONE.

[108] Baker M., Negus D., Raghunathan D., Radford P., Moore C., Clark G., Diggle M., Tyson J., Twycross J., Sockett R.E. (2017). Measuring and Modelling the Response of *Klebsiella pneumoniae* KPC Prey to *Bdellovibrio bacteriovorus* Predation, in Human Serum and Defined Buffer. Sci. Rep..

[109] Sun Y., Ye J., Hou Y., Chen H., Cao J., Zhou T. (2017). Predation Efficacy of *Bdellovibrio bacteriovorus* on Multidrug-Resistant Clinical Pathogens and Their Corresponding Biofilms. Jpn. J. Infect. Dis..

[110] Im H., Choi S.Y., Son S., Mitchell R.J. (2017). Combined Application of Bacterial Predation and Violacein to Kill Polymicrobial Pathogenic Communities. Sci. Rep..

[111] Westergaard J.M., Kramer T.T. (1977). *Bdellovibrio* and the Intestinal Flora of Vertebrates. Appl. Environ. Microbiol..

[112] Atterbury R.J., Hobley L., Till R., Lambert C., Capeness M.J., Lerner T.R., Fenton A.K., Barrow P., Sockett R.E. (2011). Effects of Orally Administered *Bdellovibrio bacteriovorus* on the Well-Being and *Salmonella* Colonization of Young Chicks. Appl. Environ. Microbiol..

[113] Romanowski E.G., Stella N.A., Brothers K.M., Yates K.A., Funderburgh M.L., Funderburgh J.L., Gupta S., Dharani S., Kadouri D.E., Shanks R.M.Q. (2016). Predatory Bacteria Are Nontoxic to the Rabbit Ocular Surface. Sci. Rep..

[114] Shatzkes K., Singleton E., Tang C., Zuena M., Shukla S., Gupta S., Dharani S., Onyile O., Rinaggio J., Connell N.D. (2016). Predatory Bacteria Attenuate *Klebsiella pneumoniae* Burden in Rat Lungs. mBio.

[115] Shatzkes K., Singleton E., Tang C., Zuena M., Shukla S., Gupta S., Dharani S., Rinaggio J., Kadouri D.E., Connell N.D. (2017). Examining the Efficacy of Intravenous Administration of Predatory Bacteria in Rats. Sci. Rep..

[116] Shatzkes K., Tang C., Singleton E., Shukla S., Zuena M., Gupta S., Dharani S., Rinaggio J., Connell N.D., Kadouri D.E. (2017). Effect of Predatory Bacteria on the Gut Bacterial Microbiota in Rats. Sci. Rep..

[117] Shatzkes K., Chae R., Tang C., Ramirez G.C., Mukherjee S., Tsenova L., Connell N.D., Kadouri D.E. (2015). Examining the Safety of Respiratory and Intravenous Inoculation of *Bdellovibrio bacteriovorus* and *Micavibrio aeruginosavorus* in a Mouse Model. Sci. Rep..

[118] Willis A.R., Moore C., Mazon-Moya M., Krokowski S., Lambert C., Till R., Mostowy S., Sockett R.E. (2016). Injections of Predatory Bacteria Work Alongside Host Immune Cells to Treat *Shigella* Infection in Zebrafish Larvae. Curr. Biol. CB.

[119] Torraca V., Mostowy S. (2018). Zebrafish Infection: From Pathogenesis to Cell Biology. Trends Cell Biol..

[120] Gomes M.C., Mostowy S. (2020). The Case for Modeling Human Infection in Zebrafish. Trends Microbiol..

[121] Howe K., Clark M.D., Torroja C.F., Torrance J., Berthelot C., Muffato M., Collins J.E., Humphray S., McLaren K., Matthews L. (2013). The Zebrafish Reference Genome Sequence and Its Relationship to the Human Genome. Nature.

[122] Meijer A.H., Spaink H.P. (2011). Host-Pathogen Interactions Made Transparent with the Zebrafish Model. Curr. Drug Targets.

[123] Russo R., Kolesnikova I., Kim T., Gupta S., Pericleous A., Kadouri D.E., Connell N.D. (2018). Susceptibility of Virulent *Yersinia pestis* Bacteria to Predator Bacteria in the Lungs of Mice. Microorganisms.

[124] Boileau M.J., Mani R., Breshears M.A., Gilmour M., Taylor J.D., Clinkenbeard K.D. (2016). Efficacy of *Bdellovibrio bacteriovorus* 109J for the Treatment of Dairy Calves with Experimentally Induced Infectious Bovine Keratoconjunctivitis. Am. J. Vet. Res..

[125] Boileau M.J., Clinkenbeard K.D., Iandolo J.J. (2011). Assessment of *Bdellovibrio bacteriovorus* 109J Killing of *Moraxella bovis* in an in Vitro Model of Infectious Bovine Keratoconjunctivitis. Can. J. Vet. Res. Rev. Can. Rech. Vet..

[126] Romanowski E.G., Brothers K.M., Calvario R.C., Stella N.A., Kim T., Elsayed M., Kadouri D.E., Shanks R.M.Q. (2024). Predatory Bacteria Prevent the Proliferation of Intraocular *Serratia marcescens* and Fluoroquinolone-Resistant *Pseudomonas aeruginosa*. Microbiology.

[127] Rendulic S., Jagtap P., Rosinus A., Eppinger M., Baar C., Lanz C., Keller H., Lambert C., Evans K.J., Goesmann A. (2004). A Predator Unmasked: Life Cycle of *Bdellovibrio bacteriovorus* from a Genomic Perspective. Science.

[128] Monnappa A.K., Dwidar M., Seo J.K., Hur J.-H., Mitchell R.J. (2014). *Bdellovibrio bacteriovorus* Inhibits *Staphylococcus aureus* Biofilm Formation and Invasion into Human Epithelial Cells. Sci. Rep..

[129] Waso M., Reyneke B., Havenga B., Khan S., Khan W. (2021). Insights into *Bdellovibrio* spp. Mechanisms of Action and Potential Applications. World J. Microbiol. Biotechnol..

[130] Zhou Y., Chen H., Jiang H., Yao Q., Zhu H. (2023). Characteristics of a Lipase ArEstA with Lytic Activity against Drug-Resistant Pathogen from a Novel Myxobacterium, Archangium *Lipolyticum* sp.. Nov. Front. Microbiol..

[131] Hobley L., Summers J.K., Till R., Milner D.S., Atterbury R.J., Stroud A., Capeness M.J., Gray S., Leidenroth A., Lambert C. (2020). Dual Predation by Bacteriophage and *Bdellovibrio bacteriovorus* Can Eradicate *Escherichia coli* Prey in Situations Where Single Predation Cannot. J. Bacteriol..

[132] Marine E., Milner D.S., Lambert C., Sockett R.E., Pos K.M. (2020). A Novel Method to Determine Antibiotic Sensitivity in *Bdellovibrio bacteriovorus* Reveals a DHFR-Dependent Natural Trimethoprim Resistance. Sci. Rep..

